# A Study on the Effect of Graphene in Enhancing the Electrochemical Properties of SnO_2_-Fe_2_O_3_ Anode Materials

**DOI:** 10.3390/ma15227947

**Published:** 2022-11-10

**Authors:** Guanglin Zhu, Bo Gao, Ying Zhang, Zeyuan Shi, Zongbin Li, Ganfeng Tu

**Affiliations:** 1 Key Laboratory for Ecological Metallurgy of Multimetallic Mineral (Ministry of Education), Northeastern University, Shenyang 110819, China; 2 Key Laboratory for Anisotropy and Texture of Materials (Ministry of Education), School of Materials Science and Engineering, Northeastern University, Shenyang 110819, China

**Keywords:** transition metal oxide, rGO, specific surface area, discharge capacity, cycle stability

## Abstract

To enhance the conductivity and volume expansion during the charging and discharging of transition metal oxide anode materials, rGO-SnO_2_-Fe_2_O_3_ composite materials with different contents of rGO were prepared by the in situ hydrothermal synthesis method. The SEM morphology revealed a sphere-like fluffy structure, particles of the 0.4%rGO-10%SnO_2_-Fe_2_O_3_ composite were smaller and more compact with a specific surface area of 223.19 m^2^/g, the first discharge capacity of 1423.75 mAh/g, and the specific capacity could be maintained at 687.60 mAh/g even after 100 cycles. It exhibited a good ratio performance and electrochemical reversibility, smaller charge transfer resistance, and contact resistance, which aided in lithium-ion transport. Its superior electrochemical performance was due to the addition of graphene, which made the spherical particle size distribution more uniform, effectively lowering the volume expansion during the process of charging and discharging and improving the electrochemical cycle stability of the anode materials.

## 1. Introduction

The acceleration of social progress and industrialization will create an increasingly high demand for energy resources. At present, the commercial lithium-ion battery anode material is mainly graphite, which has a small theoretical specific capacity (372 mAh/g) and hence cannot meet the power supply demand of large equipment [1,2,3]. Therefore, the development of lithium-ion battery anode materials with high specific capacity and stable cycle performance is a relevant research area. Compared with traditional graphite anode materials, transition metal oxide materials have a higher theoretical specific capacity [4,5,6]. For instance, Fe_2_O_3_ and SnO_2_ have a theoretical specific capacity of approximately 1004 and 783 mAh/g, respectively. These materials also have the advantages of low working potential, simple charging and discharging cycles, and abundant reserves, which makes them potential candidates [7,8,9]. However, in practical application, there are some drawbacks, such as volume expansion, low conductivity, and poor cycle stability [10,11,12,13,14,15], which might affect the battery life. Structural modification and carbon material coating treatment are the commonly adopted methods to improve its electrochemical performance [16,17,18].

Cui et al. [19] used a template method to prepare SnO_2_@Fe_2_O_3_ double-shell hollow spheres. For a current density of 100 mA/g, the specific discharge capacity decreased to 464 mAh/g after 46 cycles while increased to 1043 mAh/g after 190 cycles, showcasing better electrochemical performance. Chai et al. [20] used glucose as a carbon source and ferric nitrate as an iron source to obtain anode materials by the one-step hydrothermal method followed by argon atmosphere annealing. The material possessed high crystallinity and good thermal stability. At 0.1 A/g current density, the specific capacity was 826 mAh/g after 20 cycles, and the coulomb efficiency reached 99%. Lv et al. [21] prepared carbon-coated α-Fe_2_O_3_ nanostructured anode materials by simple pyrolysis deposition of ferrocene on stainless steel substrates. After 300 cycles at a current density of 500 mA/g, it could still retain a high reversible specific capacity of 1138 mAh/g.

Due to the unique structure of graphene, it can show good electrochemical performance when used as the anode material of lithium-ion batteries [22,23,24,25,26]. The loss of oxygen-containing functional groups increases the spacing between graphene layers, which is conducive to Li^+^ embedding and removal, alleviating volume expansion [27,28,29,30,31,32,33,34,35]. Zhou et al. [36] prepared Fe_2_O_3_/graphene nano-sheets by a simple and effective spray drying method, which showed a specific capacity of 711 mAh/g after 50 cycles of charging and discharging, and the chemical properties were quite improved. Qin et al. [37] prepared porous Fe_2_O_3_/N-doped graphene (Fe_2_O_3_/N-rGO) anode materials by a one-step in situ hydrothermal growth method. The porous structure and the introduction of curly GO greatly improved the electrochemical performance of the composite electrode, and the discharge-specific capacity was maintained at 1072.5 mAh/g after 100 cycles. Yang et al. [38] introduced a method for fabricating graphene-encapsulated metal oxides (GE-MO) via the co-assembly of negatively charged GO and positively charged oxide nanoparticles. The process was driven by the mutual electrostatic interaction of the oppositely charged particles, followed by chemical reduction generating GE-MO. The detection results showed that the electrochemically active Co_3_O_4_ nanoparticle coated with graphene (GE-Co_3_O_4_) exhibited a high reversible specific capacity of 1100 mAh/g during the first 10 cycles, and the reversible specific capacity could be maintained at 1000 mAh/g even after 130 cycles, showing excellent cycling performance. Therefore, it exhibited great potential as an anode material for lithium storage. Lian et al. [39] developed a gas-liquid interface synthesis method to prepare nano SnO_2_/graphene composites. The SnO_2_ nanoparticles could be uniformly distributed on the graphene substrate and showed a reversible specific capacity of 1304 mAh/g at a current density of 100 mA/g. Zhou et al. [40] synthesized Sn nanoparticles/graphene composites as anode materials for lithium-ion batteries and graphene nano-sheets as a reducing agent and support layer. In the G/Sn/G layer superposition structure, the large area and elastic space were provided by graphene sheets, and the Sn nanoparticles showed excellent electrical conductivity [41,42,43,44]. The material exhibited good electrochemical performance and cyclic stability. Wang et al. [45] combined 3D hydrogels with nitrogen-containing graphene. This graphene lamellar structure had a dense microstructure and high density, and a single graphene lamellar could provide conductive pathways for electrons and more edges to enhance Li^+^ embedding. Hu et al. [46] synthesized monohybrids by wrapping hollow SnO_2_ nanospheres with GO. This compound exhibited excellent electrochemical performance with a high reversible specific capacity of 1107 mAh/g after 100 cycles at a current density of 0.1 A/g.

To enhance the conductivity and volume expansion in the charging and discharging cycles of transition metal oxide anode materials, this paper employed the in situ hydrothermal synthesis method to prepare rGO-SnO_2_-Fe_2_O_3_ composite materials. The phase composition and microstructure were determined by XRD and SEM. The charge and discharge performance, cycle performance, rate performance, and AC impedance of the materials were tested. The physical and chemical properties and internal mechanisms of the composite materials were explored.

## 2. Experiment

### 2.1. Preparation of Anode Materials

A certain amount of graphene oxide was added to deionized water and ultrasonic dispersion for 1 h, then the percentage of tin chloride and ferric chloride was dissolved. Then, sodium hydroxide solution (1 mol/L) was added during the mixing process until the solution pH reached approximately 9. After stirring for 4 h, the mixture was shifted to the high-pressure reaction kettle (Sinopharm Chemical Reagent Co., Ltd., Shenyang, China) for 4 h under 100 °C. It was further cooled to room temperature and allowed to stand for 8 h, washed, dried at 60 °C for 24 h, and lastly, vacuum roasted at 400 °C for 4 h to obtain the rGO-SnO_2_-Fe_2_O_3_ composite metal oxide. The Sn content was calculated as SnO_2_, the mass percentage was fixed at 10%, and the graphene content was varied as 0.2%, 0.4%, 0.6%, and 0.8%, respectively.

### 2.2. Electrode Sheet Preparation

The prepared anode material was used as the active material, acetylene black was used as the conductive agent, and PVDF was used as the binder. The mass ratio was fixed as 8:1:1. N-methyl pyrrolidone (NMP) was used as the solvent to prepare the slurry. After coating, vacuum drying at 80 °C for 12 h, and stamping, the electrode sheet was ready for use.

### 2.3. The Battery Assembly

The electrochemical performance tests of electrode materials were executed by using the half-cell test system, the prepared electrode sheet as the anode, the lithium sheet as the counter electrode and auxiliary electrode, the polypropylene microporous membrane (Celgard-2400, Charlotte, NC, USA) as the diaphragm, and 1 mol/L LiPF_6_/ethylene carbonate (EC) + dimethyl carbonate (DMC) (volume ratio was 1:1) mixture solution was used as the electrolyte. The battery was assembled according to a certain sequence of a positive shell, electrode sheet, diaphragm, lithium sheet, gasket, spring sheet, and negative shell. During the assembly process, the electrolyte was added to both sides of the diaphragm.

### 2.4. Characterizations

The surface morphology of the anode material was observed and analyzed by a SSX-550 tungsten filament analytical scanning electron microscope (Shimadzu Corporation, Kyoto, Japan).

A X ‘Pert Pro MPD diffractometer PW3040/60 manufactured by Koninklijke Philips N.V., Amsterdam, The Netherlands was used. The Kα of the Cu target was the radiation source (wavelength 1.540598 A), the working voltage was 40 kV, the sweep speed was set as 6°/min, and the sweep range was 10–90°.

The surface area tester (Micromeritics ASAP 2020) produced by Micromeritics (Pudong, China) was used to calculate the specific surface area of the sample by Brunauer–Emmett–Teller (BET) isotherm method.

### 2.5. Electrochemical Performance Test

A constant current charge/discharge test was carried out following the steps of static-constant current discharge-static-constant current charge-constant voltage charge. The test voltage window was set as 0.01–3 V. The charge and discharge current values were calculated from the theoretical specific capacity and the mass of the active substance.

The instrument used for the cyclic voltammetry test was the CHI660D electrochemical workstation of Shanghai Chenhua Company, Shanghai, China. The test conditions were as follows: scan rate of 0.1 mV/s, the voltage scan range of 0–3.0 V.

In the Electrochemical impedance spectroscopy test, the frequency range was 0.01 Hz–1 MHz, and the voltage vibration amplitude was 5 mV.

## 3. Results and Discussion

### 3.1. Microstructure Morphology and Specific Surface Area

**Figure 1:**
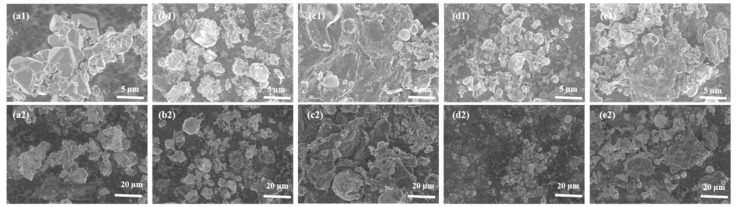
SEM images of rGO-SnO_2_-Fe_2_O_3_ composites. (**a1**) High magnification image of 10%SnO_2_-Fe_2_O_3_; (**a2**) Low magnification image of 10%SnO_2_-Fe_2_O_3_; (**b1**) High magnification image of 0.2%rGO-10%SnO_2_-Fe_2_O_3_; (**b2**) Low magnification image of 0.2%rGO-10%SnO_2_-Fe_2_O_3_; (**c1**) High magnification image of 0.4%rGO-10%SnO_2_-Fe_2_O_3_; (**c2**) Low magnification image of 0.4%rGO-10%SnO_2_-Fe_2_O_3_; (**d1**) High magnification image of 0.6%rGO-10%SnO_2_-Fe_2_O_3_; (**d2**) Low magnification image of 0.6%rGO-10%SnO_2_-Fe_2_O_3_; (**e1**) High magnification image of 0.8%rGO-10%SnO_2_-Fe_2_O_3_; (**e2**) Low magnification image of 0.8%rGO-10%SnO_2_-Fe_2_O_3_.

Figure 1 shows the SEM of the rGO-SnO_2_-Fe_2_O_3_ composite oxide samples with varying graphene contents, and the corresponding specific surface area is listed in Table 1. The 10%SnO_2_-Fe_2_O_3_ composite oxide exhibited an irregular structure. After adding graphene, the rGO-10%SnO_2_-Fe_2_O_3_ composite materials exhibited a globular fluffy structure, and the granular complex was distributed on the surface of the gossamer graphene, in between the lamellae (Figure 1). The gap between the particles could be filled with the later added conductive agent and binder. This addition, in turn, helped speed up the electron transmission, and the graphene with small resistance could inhibit the volume expansion effect during the charging and discharging process. By comparing the SEM images of rGO-SnO_2_-Fe_2_O_3_ composites with varying graphene contents, it could be found that 0.4%rGO-10%SnO_2_-Fe_2_O_3_ composites had the best microstructure morphology with smaller and more spherical particles, which are closely packed as well. The complex particles were evenly dispersed and tightly fixed on the curled, wrinkled surface of graphene, and the corresponding specific surface area was 223.19 m^2^/g. When the amount of graphene was too large, as in 0.8%rGO-10%SnO_2_-Fe_2_O_3_, the agglomeration phenomenon was obvious, leading to a large number of sheet structures, and the specific surface area was 204.33 m^2^/g, which was not ideal for the transport of lithium ions during the charging and discharging process. The addition of graphene avoided the phenomenon of the anode material getting expanded or even the outer layer falling off during the charging or discharging process of lithium-ion batteries.

### 3.2. Phase Composition Analysis

**Figure 2:**
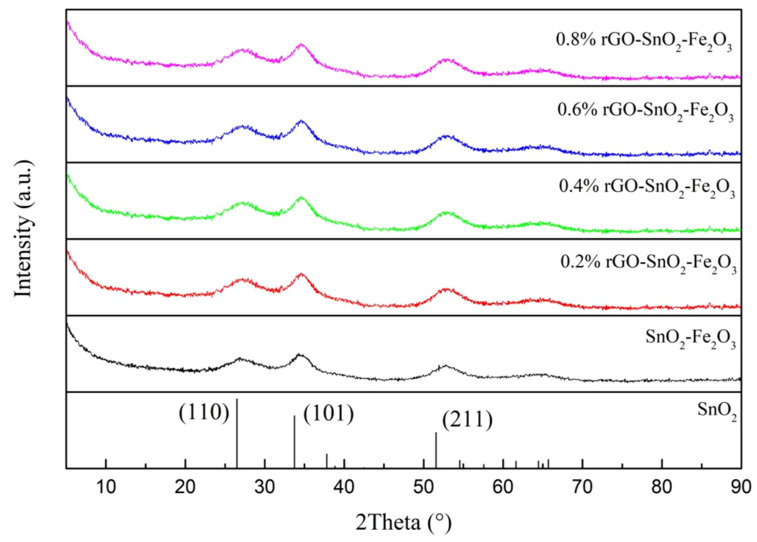
XRD patterns of rGO-SnO_2_-Fe_2_O_3_ composites.

The XRD patterns of rGO-SnO_2_-Fe_2_O_3_ composites with different graphene contents are shown in Figure 2. The peak patterns of rGO-SnO_2_-Fe_2_O_3_ composite compounds with different graphene contents were similar (Figure 2). Only the characteristic diffraction peaks of SnO_2_ are shown in the XRD pattern, suggesting that the main phase of rGO-SnO_2_-Fe_2_O_3_ composites is SnO_2_. There were no characteristic peaks corresponding to graphene and Fe_2_O_3_ in the XRD pattern, possibly because the content of graphene was low, and iron oxide might exist in an amorphous form, or enter the lattice inside of tin oxide during the recombination process to form the solid solution.

### 3.3. Cyclic Performance Analysis

**Figure 3:**
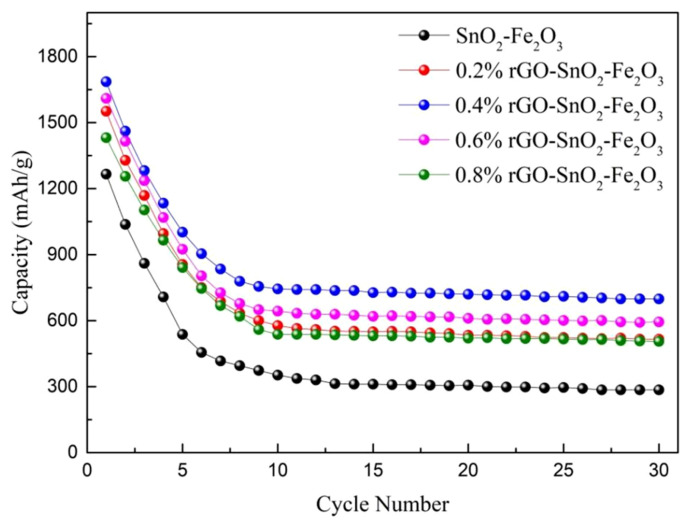
Cyclic performance of rGO-SnO_2_-Fe_2_O_3_ composites.

Figure 3 shows the cycle performance curves of rGO-SnO_2_-Fe_2_O_3_ complexes with different graphene contents at a current density of 100 mA/g. As shown in Figure 3, the first discharge-specific capacity of rGO-SnO_2_-Fe_2_O_3_ composites increased significantly with graphene addition, indicating that the addition could improve the transport and diffusion of lithium ions, and the specific capacity of the material was improved. With graphene content increasing, capacity firstly increased and then decreased, and the capacity of 0.4%rGO-SnO_2_-Fe_2_O_3_ reached the maximum. The specific capacity loss in the first cycle was due to the formation of solid electrolyte interface film [47,48]. It is also worth noting that the cycling performance of rGO-SnO_2_-Fe_2_O_3_ composite was significantly improved with an increased amount of graphene compared with that of 10% SnO_2_-Fe_2_O_3_ composite oxide. Among them, the 0.4%rGO-10%SnO_2_-Fe_2_O_3_ anode material showed the best cycling performance, the first discharge-specific capacity was approximately 1423.75 mAh/g, and the cycle stability was at its best. The discharge-specific capacity was retained at approximately 839.76 mAh/g after 10 cycles. After 50 cycles, the discharge-specific capacity could be kept close to 744.05 mAh/g, which was mainly due to the more uniform distribution of spherical particles in the microstructure of 0.4%rGO-10%SnO_2_-Fe_2_O_3_. When the graphene content was increased to 0.8%, the cycling performance of 0.8%rGO-10%SnO_2_-Fe_2_O_3_ decreased significantly, which might be due to the high agglomeration tendency of material particles caused by the excessive amount of graphene.

**Figure 4:**
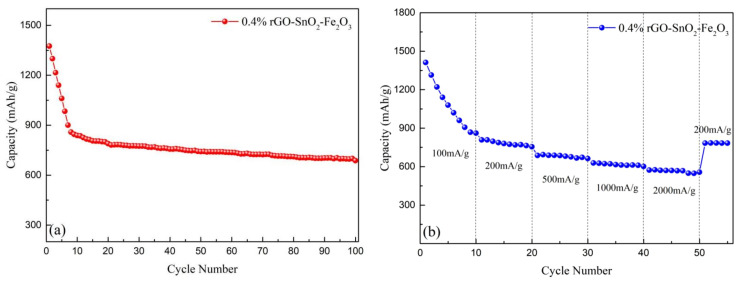
Cyclic performance diagrams of the 0.4%rGO-10SnO_2_-Fe_2_O_3_ anode material. (**a**) A total of 100 discharge cycles; (**b**) Magnification curve.

To explore the cycle life and stability of the 0.4%rGO-10%SnO_2_-Fe_2_O_3_ anode material, 100 cycles of constant current charge and discharge, and constant current at different rates are shown in Figure 4. According to the test results in Figure 4a, the discharge specific capacity of the 0.4%rGO-10%SnO_2_-Fe_2_O_3_ anode material was maintained at approximately 687.60 mAh/g even after 100 cycles. In subsequent tests, although the specific capacity decreased to a small extent, the change was very gentle, indicating that the material had relatively stable cycling performance. It could be seen from Figure 4b, that after different rate tests, the specific capacity of the 0.4%rGO-10%SnO_2_-Fe_2_O_3_ anode material decreased with an increase in the rate, but the overall decline rate was relatively slow. When the rate was 0.2 C, the discharge capacity was 763.82 mAh/g; however, at a 2 C rate, the specific capacity was 556.77 mAh/g. The specific capacity was 556.77 mAh/g when the rate was restored to 0.2 C, while the specific discharge capacity was not reduced but increased to 651 mAh/g. This result indicates that the overall performance of the material was improved to a certain extent due to the mutual synergistic effect of the combination of different components. Moreover, after a period of charging and discharging, the infiltration of the electrolyte and the volume change led to a more stable structure of the anode material, and thus its discharge-specific capacity was improved.

### 3.4. Charge and Discharge Curve Analysis

**Figure 5:**
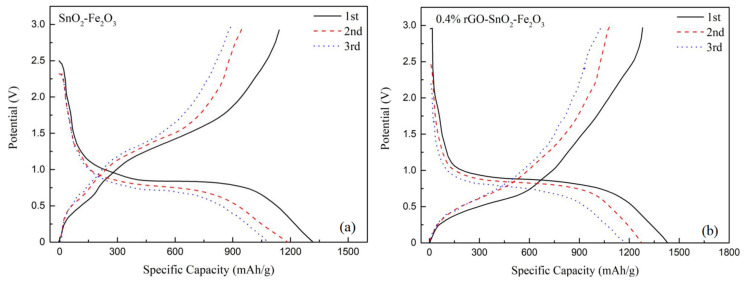
The first three-time capacity-voltage curves. (**a**) 10%SnO_2_-Fe_2_O_3_; (**b**) 0.4%rGO-10%SnO_2_-Fe_2_O_3_.

The first three charge and discharge tests of 10%SnO_2_-Fe_2_O_3_ and 0.4%rGO-10%SnO_2_-Fe_2_O_3_ anode materials were carried out, as shown in Figure 5. According to Figure 5a, in the first discharge process of the 10%SnO_2_-Fe_2_O_3_ sample, there was a flat and long discharge platform at approximately 0.8 V, and a relatively slow charging voltage platform at 1.5–1.8 V appeared in the first charging curve. Similarly, from Figure 5b, it can be seen that during the first discharge process of the 0.4%rGO-10%SnO_2_-Fe_2_O_3_ sample, there was a short platform approximately 2.0 V, a flat long platform approximately 0.8–0.9 V, and a slow charging platform between 1.0 and 1.5 V in the first charging curve. The first discharge exhibited the largest specific capacity, and the second turn had the smallest specific capacity loss. The second and third charge and discharge curves showed a gentle variation trend, higher coincidence, and better electrochemical reversibility, which can be attributed to the fact that the addition of graphene made the distribution of the spherical particles more uniform, leading to an alleviated volume expansion effect during the process of charge and discharge thereby improving the electrochemical stability of anode materials.

### 3.5. Cyclic Voltammetry Curve Analysis

**Figure 6:**
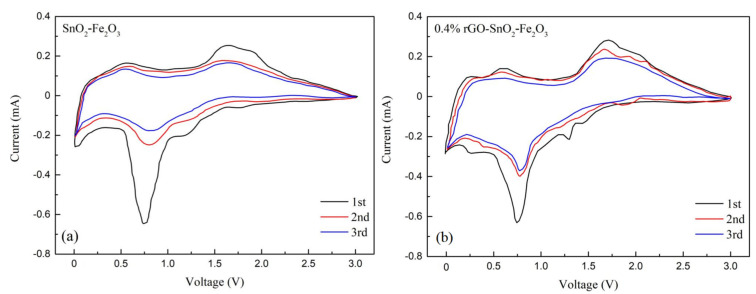
Cyclic voltametric curve. (**a**) 10%SnO_2_-Fe_2_O_3_; (**b**) 0.4% rGO-10%SnO_2_-Fe_2_O_3_.

Cyclic voltametric curves of 10%SnO_2_-Fe_2_O_3_ and 0.4%rGO-10%SnO_2_-Fe_2_O_3_ are shown in Figure 6. As can be seen from Figure 6a, the reduction peak of 10%SnO_2_-Fe_2_O_3_ in the first turn appeared near 0.78 V, which corresponds to the conversion of Fe^3+^ to Fe^0^. In addition, there was a smaller reduction peak located at 1.3 V, which was assigned to the reduction of Sn^4+^ to Sn^2+^. However, the peak was of significantly low intensity. The oxidation peak in the first cycle appeared at 1.7–2.2 V, which corresponds to the transformation of Fe^0^ to Fe^3+^ and Sn^2+^ to Sn^4+^. In the following two cycles, the intensity of the oxidation peak decreased slightly, and the overlap of cyclic voltammetry curves was good. Figure 6b shows the charging and discharging cyclic voltammetry curves of 0.4%rGO-10%SnO_2_-Fe_2_O_3_ for the first three times. The reduction peak of 0.4%rGO-10%SnO_2_-Fe_2_O_3_ in the first turn appeared near 0.7 V, which corresponded to the conversion of Fe^3+^ to Fe^0^. In addition, there was a smaller reduction peak located at 1.3 V attributable to Sn^4+^ to Sn^2+^ conversion [49,50,51]. In the following two turns, the position of the reduction peak from Fe^3+^ to Fe^0^ did not move, but the peak intensity was significantly weakened due to the formation of solid electrolyte interface film (SEI film) [52,53], and the reduction peak from Sn^4+^ to Sn^2+^ disappeared. The oxidation peaks of the first cycle appeared at 1.7 V and 2.0 V, corresponding to the transformation of Fe^0^ to Fe^3+^ and Sn^2+^ to Sn^4+^. The cyclic voltammetry curves overlapped well, suggesting higher reversibility and better stability.

### 3.6. AC Impedance Analysis

**Figure 7:**
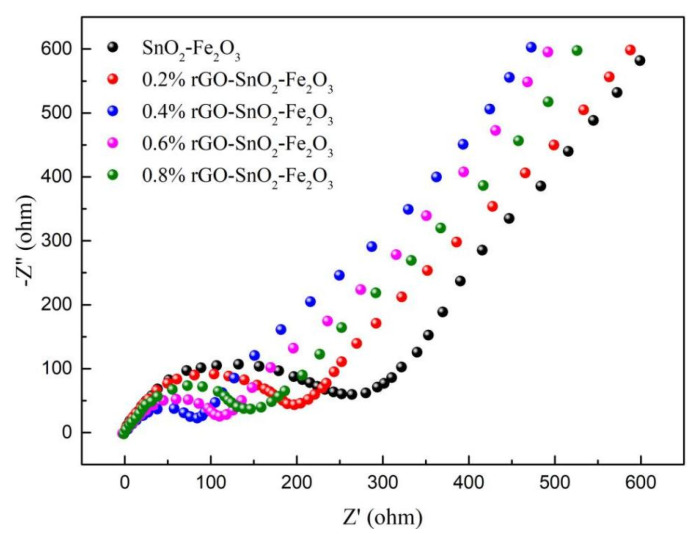
AC impedance diagrams of rGO-SnO_2_-Fe_2_O_3_ composites.

The AC impedance spectra of rGO-SnO_2_-Fe_2_O_3_ composites with different graphene contents are shown in Figure 7. The AC impedance spectra of all samples consisted of a semicircle in the high-frequency region and a straight line in the low-frequency region. The radius of the semicircle represented the electron transfer resistance at the electrode/solution interface, which was also the charge transfer impedance at the electrode interface and corresponded to the contact resistance of the anode material [54]. The slope of the line represented the mobility of lithium ions inside the electrode, namely the Warburg impedance, which could be related to the charge transfer resistance [55,56,57,58]. Figure 7 showed that the resistance value of composite material increases in the order: 0.4%rGO-10%SnO_2_-Fe_2_O_3_, 0.6%rGO-10%SnO_2_-Fe_2_O_3_, 0.8%rGO-10%SnO_2_-Fe_2_O_3_, 0.2%rGO-10%SnO_2_-Fe_2_O_3_ and 10%SnO_2_-Fe_2_O_3_. After graphene addition, the resistance of the material decreased, which was closely related to the effect of graphene on the microstructure. Among them, when the graphene content was 0.4%, the 0.4%rGO-10%SnO_2_-Fe_2_O_3_ anode material possessed the smallest semi-circuit radius, indicating that it had the smallest contact resistance and charge transfer resistance, which was conducive to the transport of lithium ions and was consistent with the cycling performance results. With the amount of graphene increasing, as in 0.8%rGO-10%SnO_2_-Fe_2_O_3_, the charge transfer resistance was increased due to the agglomeration phenomenon and lower specific surface area.

## 4. Conclusions

In this paper, rGO-SnO_2_-Fe_2_O_3_ anode materials were prepared by using SnO_2_-Fe_2_O_3_ as the matrix and adding graphene oxide in varying contents. The structural characterization and electrochemical performance of the above materials were investigated, and the following conclusions were drawn: (1) The phase structure of the rGO-SnO_2_-Fe_2_O_3_ composite was SnO_2_, and the morphology was a globular fluffy structure. The granular complex was distributed on the surface and between the lamellae of the bubble graphene. The morphology of 0.4%rGO-10%SnO_2_-Fe_2_O_3_ composite was the best, and the spherical particles were smaller and more in numbers. The spherical particles were more closely packed, and the specific surface area was 223.19 m^2^/g. (2) The cycle performance of the 0.4%rGO-10%SnO_2_-Fe_2_O_3_ anode material was the best, the first discharge-specific capacity was 1423.75 mAh/g, and after 100 cycles, the capacity was maintained at approximately 687.60 mAh/g. The results of different rate tests showed that the specific capacity of the 0.4%rGO-10%SnO_2_-Fe_2_O_3_ anode material decreased with an increase in the rate, but the overall rate of decline was relatively slow. (3) 0.4%rGO-10%SnO_2_-Fe_2_O_3_ exhibited better electrochemical reversibility, smaller contact resistance, and charge transfer resistance. All these characteristics were beneficial to the transport of lithium ions and were consistent with the cycling performance results. (4) The excellent electrochemical performance of 0.4%rGO-10%SnO_2_-Fe_2_O_3_ can be attributed to the addition of graphene, which made the size distribution of spherical particles more uniform, effectively alleviating the volume expansion phenomenon during the process of charging and discharging. This has further enhanced the electrochemical cycle stability of anode materials. It can be seen from the experimental results that the addition of graphene can improve the specific capacity and cycle stability. However, it has no significant effect on the capacity attenuation in the first lap, which will be paid more attention in the later research.

## Figures and Tables

**Table 1:** Specific surface area of rGO-SnO_2_-Fe_2_O_3_ composite oxides.

Samples	SnO_2_-Fe_2_O_3_	0.2%rGO- SnO_2_-Fe_2_O_3_	0.4%rGO- SnO_2_-Fe_2_O_3_	0.6%rGO- SnO_2_-Fe_2_O_3_	0.8%rGO- SnO_2_-Fe_2_O_3_
S_BET_(m^2^/g)	145.43	183.67	223.19	216.99	204.33

## Data Availability

All data generated and analyzed during this study are included in this article.
